# ACE Gene I/D Polymorphism and Acute Pulmonary Embolism in COVID19 Pneumonia: A Potential Predisposing Role

**DOI:** 10.3389/fmed.2020.631148

**Published:** 2021-01-21

**Authors:** Cecilia Calabrese, Anna Annunziata, Antonietta Coppola, Pia Clara Pafundi, Salvatore Guarino, Valentina Di Spirito, Valeria Maddaloni, Nicola Pepe, Giuseppe Fiorentino

**Affiliations:** ^1^Department of Translational Medical Sciences, University of Campania “Luigi Vanvitelli”, Naples, Italy; ^2^Department of Intensive Care, A.O.R.N. Ospedali dei Colli, Naples, Italy; ^3^Department of Advanced Medical and Surgical Sciences, University of Campania “Luigi Vanvitelli”, Naples, Italy; ^4^Department of Radiodiagnostic, A.O.R.N. Ospedali dei Colli, Naples, Italy; ^5^Molecular Genomics Lab, Chemical Biochemistry Unit, A.O.R.N. Ospedali dei Colli, Naples, Italy

**Keywords:** COVID19 pneumonia, pulmonary embolism, ace gene, polymorphism, angiotensin II

## Abstract

Most recent studies have stressed a high risk of thromboembolism in patients with SARS-CoV-2 infection, particularly in those with severe COVID-19 pneumonia. Counterbalance between angiotensin-converting-enzyme (ACE) and ACE2 activities in COVID-19 disease may be crucially involved in the thrombo-inflammatory process. Currently, no study has investigated ACE I/D polymorphism involvement in COVID-19 disease complicated by pulmonary embolism, hence the aim of the present pilot study. This is a retrospective, single-center observational case-control study, conducted at the Sub-Intensive Care Unit of A.O.R.N. Ospedali dei Colli, Cotugno Hospital, Naples (Italy). We included 68 subjects with severe/critical COVID-19 pneumonia. COVID-19 patients were divided according to occurrence of PE (PE+, *n* = 25) or absence of thromboembolic complications (PE−, *n* = 43). Assessment of ACE I/D polymorphisms showed a statistically significant difference between PE+ and PE− patients (*p* = 0.029). Particularly, prevalence of D/D homozygous polymorphism was significantly higher in PE+ COVID-19 patients than in PE− (72 vs. 46.5%; *p* = 0.048), while heterozygote I/D polymorphism was significantly lower expressed in PE+ patients than in PE− (16 vs. 48.8%; *p* = 0.009). Computed tomographic pulmonary angiography showed predominantly mono/bilateral sub-segmental embolisms. In conclusion, our findings let us hypothesize a genetic susceptibility to thromboembolism in COVID-19 disease. ACE D/D polymorphism might represent a genetic risk factor, although studies on larger populations are needed.

## Introduction

Most recent studies have stressed a high risk of thromboembolism in patients affected by SARS-CoV-2 infection, particularly in those with severe COVID-19 pneumonia.

Laboratory findings from retrospective cohort studies suggest an activation of the coagulation cascade characterized by elevated levels of D-dimer and fibrinogen in association with other altered coagulation indexes (1, 2). In addition, anticoagulant therapy with low molecular weight heparin reduced mortality rates of patients affected by severe SARS-CoV2 pneumonia showing an increase either in D-dimer levels and/or high sepsis-induced coagulopathy score (3, 4).

Venous thromboembolism in COVID-19 patients has been largely reported most in Intensive Care Units, with a prevalence ranging from 17 to 69% (5–9).

Nonetheless, the pathogenesis of the increased thromboembolic risk in COVID-19 pneumonia still remains unclear. Several mechanisms have been hypothesized as involved in this process, i.e., the direct cytotoxic effect induced by the virus, the endothelial cell inflammation and the dysregulated immune response, which ultimately result in the recruitment of inflammatory cells, platelet aggregation and activation of the complement and coagulation cascade (10).

In addition, the counterbalance between angiotensin-converting enzyme (ACE) and ACE2 activities occurring in COVID-19 may play a crucial role in the thrombo-inflammatory process. ACE cleaves Angiotensin (Ang) I to produce Ang II, whilst ACE 2 converts Ang II in the protective Ang 1-7. The loss of ACE2, the receptor of the SARS-CoV2 spike protein, leaves unopposed effects of Angiotensin II, thus leading to vasoconstriction, endothelial injury, endovascular thrombosis, and increased blood volume (10, 11).

In the literature, several polymorphisms of ACE gene have been described, among which the either presence (insertion, allele I) or absence (deletion, allele D) of a 287-base pair (bp) Alu repeat sequence in intron 16. A strong association between these polymorphisms and serum levels of ACE has been reported, with D/D homozygotes having 65% more, and I/D heterozygotes 31% more ACE than I/I homozygotes (12).

Indeed, several studies have also demonstrated an association between the frequency of ACE D/D polymorphism and both the prevalence and the mortality rates of COVID-19 (13, 14).

Up to now, no study has investigated the involvement of ACE I/D polymorphism in COVID-19 complicated by pulmonary embolism, hence the aim of the present pilot study.

## Materials and Methods

### Study Design

This is a retrospective, single-center observational case-control study, conducted at the Sub-Intensive Care Unit of A.O.R.N. Ospedali dei Colli, Cotugno Hospital, Naples (Italy). We included all patients suffering from COVID-19 pneumonia hospitalized between March 20, 2020 and July 20, 2020 with suspect of pulmonary embolism.

Severe/Critical COVID-19 patients, according to the World Health Organization classification (15), were divided into two subgroups according to their either occurrence or not of pulmonary embolism (PE) during SARS-CoV2 lung infection. The diagnosis of pulmonary embolism was performed by computed tomographic pulmonary angiography and lower-limb compression ultrasonography.

We compared our COVID-19 ACE genotype frequencies with those from the Italian general population reported on https://alfred.med.yale.edu/alfred/index.asp.

All subjects provided their written informed consent to the treatment of their data for clinical and research purposes. The study was approved by the local Ethic committee of AORN Ospedali dei Colli and it is in accordance with the 1976 Declaration of Helsinki and its later amendments.

### Parameters

Upon admission, all patients were asked for collection of anamnestic and anthropometric data (age, sex, BMI, smoking habit), presence of comorbidities (systemic arterial hypertension, type 2 diabetes mellitus, cardiovascular disease, chronic obstructive pulmonary disease, deep venous thrombosis, and obesity) and medication intake (e.g., antihypertensives such as ACE inhibitors or AT1 receptor antagonists).

All patients underwent to molecular analysis for thrombophilia and cardiovascular risk factors, performed by our Hospital Central Laboratory using a Reverse Dot Blot (RDB) kit by Nuclear Laser Medicine (NLM, version 2020.10.19, CVD-14 cod. AC084, Milan). In depth, we focused on Angiotensin I Converting Enzyme (ACE I/D polymorphism) (rs1799752 SNP): I/I = Insertion in homozygosis, I/D = Insertion/Deletion, and D/D = Deletion in homozygosis.

### Statistical Analysis

Categorical variables were expressed as number and percentage whilst continuous variables by median and interquartile range, after Shapiro-Wilk test to assess for normal distribution. The differences between groups were evaluated either by the Fisher's exact test or Chi-square test, depending on the sample size. Where appropriate, Yates correction was applied to the Chi Square test (in case of cell width <5 and table “m X n”) and Dunn-Sidak correction to Fisher Exact test. For all statistical tests, we used the Stata 15.0 (StataCorp, Texas, USA) and SPSS 24.0 (SPSS INC, Chicago, IL, USA). Statistical significance was defined as *p* < 0.05 and all *p*-values were two-tailed.

## Results

### General Characteristics of the Study Population

The overall study population included 68 patients with either severe or critical COVID-19 pneumonia.

COVID-19 patients were further divided into two subgroups according to the occurrence, during hospitalization, of PE (PE+, *n* = 25) or absence of any thromboembolic complication (PE−, *n* = 43). The median age of the study population was 58.5 years [IQR: 46.3–66], and they were mainly males (69.1%). As for smoking, the 50% were non-smokers, only the 2.9% were smokers, whilst the 39.7% were past-smokers.

The most prevalent comorbidity was arterial hypertension (50%), while diabetes was present in 14.7% of patients, cardiovascular disease in the 11.8% and COPD in 7.4%.

In depth, among the 34 patients with arterial hypertension, 8 (23.5%) were on ACE inhibitors and 11 (32%) on angiotensin receptor blockers.

The comparison of demographic, clinical and laboratory characteristics between the two subgroups (PE+ vs. PE−) did not shown any statistically significant difference, except for a higher prevalence of smoking habit and an increase in serum C-reactive protein in the PE+ group.

General characteristics of the study population are described in Table 1.

**Table 1:** Baseline clinical and laboratory characteristics of the overall study population and study subgroups according to the presence of pulmonary embolism (PE+ and PE−) (*n* = 68).

**Parameter**	**Overall Population (*n* = 68)**	**PE + (*n* = 25)**	**PE – (*n* = 43)**	***P***
Age (yrs.), median [IQR]	58.5 [46.3-66]	62 [49–67.5]	57 [42–65]	0.315
Sex, *n* (%)				0.502
*M/F*	47 (69.1)/21 (30.9)	19 (76)/6 (24)	29 (68.3)/14 (31.7)	
BMI (kg/m2), median [IQR]	28 [26-30.3]	28 [27–31.5]	27 [24.5–29.2]	0.182
Obesity, *n* (%)	20 (29.4)	8 (32)	12 (27.9)	0.781
Smoking habit, *n* (%)				**0.035**
*Yes*	2 (2.9)	2 (8)	-	
*No*	34 (50)	17 (68)	22 (48.8)	
*Ex*	27 (39.7)	6 (24)	21 (51.2)	
Hypertension, *n* (%)	34 (50)	13 (52)	21 (48.8)	0.897
Diabetes, *n* (%)	10 (14.7)	3 (12)	7 (16.3)	0.859
CAD, *n* (%)	8 (11.8)	-	8 (18.6)	0.051
COPD, *n* (%)	5 (7.4)	1 (4)	4 (9.3)	0.711
Deep Venous Thrombosis, *n* (%)	1 (1.5)	1 (4)	-	0.801
Pulmonary Embolism, *n* (%)	25 (36.8)			n.a.
**Laboratory**				
CRP (mg/dL), median [IQR]	11.7 [3.4–20.3]	13.7 [11–20.5]	3.5 [0.4–13.9]	**0.014**
IL-2R (IU), median [IQR]	913 [500–1561]	932 [764–1428]	614 [275–1565.5]	0.369
IL-6 (pg/mL), median [IQR]	57 [20.7–207]	59 [25–211]	52.5 [6.6–174.9]	0.367
PT, median [IQR]	84 [71–92]	80 [66–102]	91 [75–92]	0.397
aPTT, median [IQR]	34.1 [31.7–36.6]	33.9 [30.6–36]	34.2 [32.9–43.1]	0.456
PT-INR, median [IQR]	1.17 [0.93–1.27]	1.18 [1.08–1.44]	1.06 [0.8–1.22]	0.295
D-dimer (ng/mL), median [IQR]	497 [172–3189]	2235 [246–5959]	204 [163–1186]	0.222
Fibrinoge *n* (mg/dL), median [IQR]	534 [380–756]	576 [359–842]	427. [371–734]	0.628
P/F, median [IQR]	145 [102–285]	125 [98–200]	182 [122–322]	0.121
**Therapy**				
Ace-inhibitors, *n* (%)	8 (11.8)	4 (16)	4 (9.3)	0.610
Sartans, *n* (%)	11 (16.2)	4 (16)	7 (16.3)	1.000

*PE, pulmonary embolism; M, male; F, female; BMI, Body Mass Index; CAD, Coronary Artery Disease; COPD, Chronic Obstructive Pulmonary Disease; CRP, C reactive protein; IL, interleukin; PT, prothrombin time; P/F: PaO2/FiO2; IQR, interquartile range*.

*Reference ranges: IL-2R: 223–710; IL-6: 0–5; PT: 70–125; aPTT: 25–40; PT-INR: 0.8–1.2; D-dimer: <250 ng/mL; Fibrinogen: 175–417*.

### Assessment of ACE I/D Polymorphism

Table 2 describes the allelic and genotypic frequencies of the rs1799752 SNP. We compared the genetic frequencies of the rs1799752 SNP of PE+ and PE− COVID-19 patients with the Italian control population (CP) published by the Yale Genom Databank website (https://alfred.med.yale.edu/alfred/index.asp). From Alfred database we know that 222 ACE I/D alleles were studied, with an I allele frequency of 34.2% (76 alleles) vs. 65.8% for D allele (146 alleles). So there are 13 I/I; 50 I/D; and 48 D/D in the control population. Thus, checking for Hardy-Weinberg equilibrium (HWE): PE+ *p*-value is 0.027, so HWE is not satisfied. Conversely, PE− *p*-value is 0.456 so the two populations are in HWE.

**Table 2:** Allelic and genotypic frequencies and Hardy Weinberg Equilibrium test for ACE I/D polymorphism rs1799752 in PE+ and PE− COVID-19 patients, and in the control population.

	**Control Population**	**COVID-19 PE+**	**COVID-19 PE−**	***p***
**Parameter**	**(*n* = 222)**	**(*n* = 25)**	**(*n* = 43)**	
Allele, *n* (%)				0.500
*I*	76 (34.2)	7 (24.1)	23 (35.9)	
*D*	146 (65.8)	22 (75.9)	41 (64.1)	
Genotype, *n* (%)				**0.029**
*I/I*	13 (11.7)	3 (12)	2 (4.7)	
*I/D*	50 (45)	4 (16)	21 (48.8)	
*D/D*	48 (43.3)	18 (72)	20 (46.5)	
HWE		0.027	0.456	

^*^Values are expressed as absolute numbers (%). p-values were estimated with Fisher Exact Test with Dunn-Sidak correction.

^**^HWE: Hardy Weinberg Equilibrium; PE: Pulmonary Embolism; ACE: Angiotensin I Converting Enzyme.

As for allele differences between the three subgroups (controls, PE+ and PE− COVID-19 patients) no significant difference was found at Fisher's test (*p* = 0.500), whilst there was a significant difference between groups as for genotypes (*p* = 0.029).

Looking at differences among genotypes between the three subgroups, the following results were obtained. Comparison between CP and PE+ genotypes (13 50 48; 3 4 18) showed a *p*-value = 0.013, whilst CP vs. PE− (13 50 48; 2 21 20) a p-value = 0.447 and, finally, PE+ vs. PE− (3 4 18; 2 21 20) a *p*-value = 0.015. Sorting into ascending and using Dunn-Sidak thresholds order we found a difference between CP and PE+ (0.013 < 0.017) and between PE+ and PE− (0.015 < 0.025), whilst no difference emerged as between CP and PE− (0.447 > 0.050).

A significantly higher prevalence of D/D homozygous polymorphism in PE+ COVID-19 patients than in PE− (72% vs. 46.5%; *p* = 0.048) was observed, while heterozygote I/Dpolymorp hism presented a significantly lower expression in PE+ patients rather than in PE− (16% vs. 48.8%; *p* = 0.009). No significant difference instead emerged with regard to homozygote polymorphism I/I (*p* = 0.349). Computed tomographic pulmonary angiography showed predominantly mono- and bilateral sub-segmental embolisms (Figure 2 and Supplementary Figure 1).

**Figure 1:**
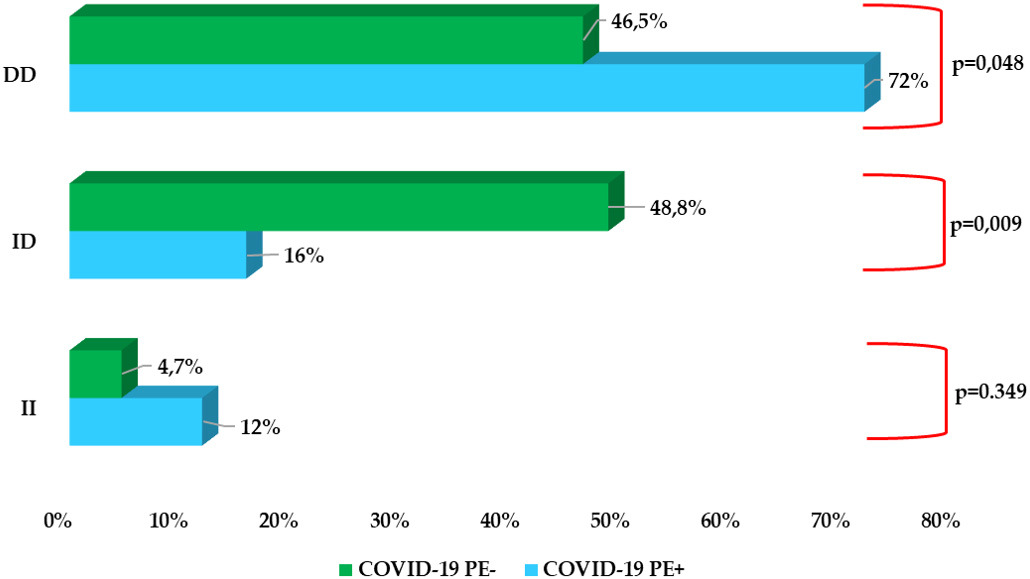
Prevalence of ACE I/D polymorphism rs1799752 in COVID-19 PE+ subgroup vs COVID-19 PE− subgroup.

**Figure 2:**
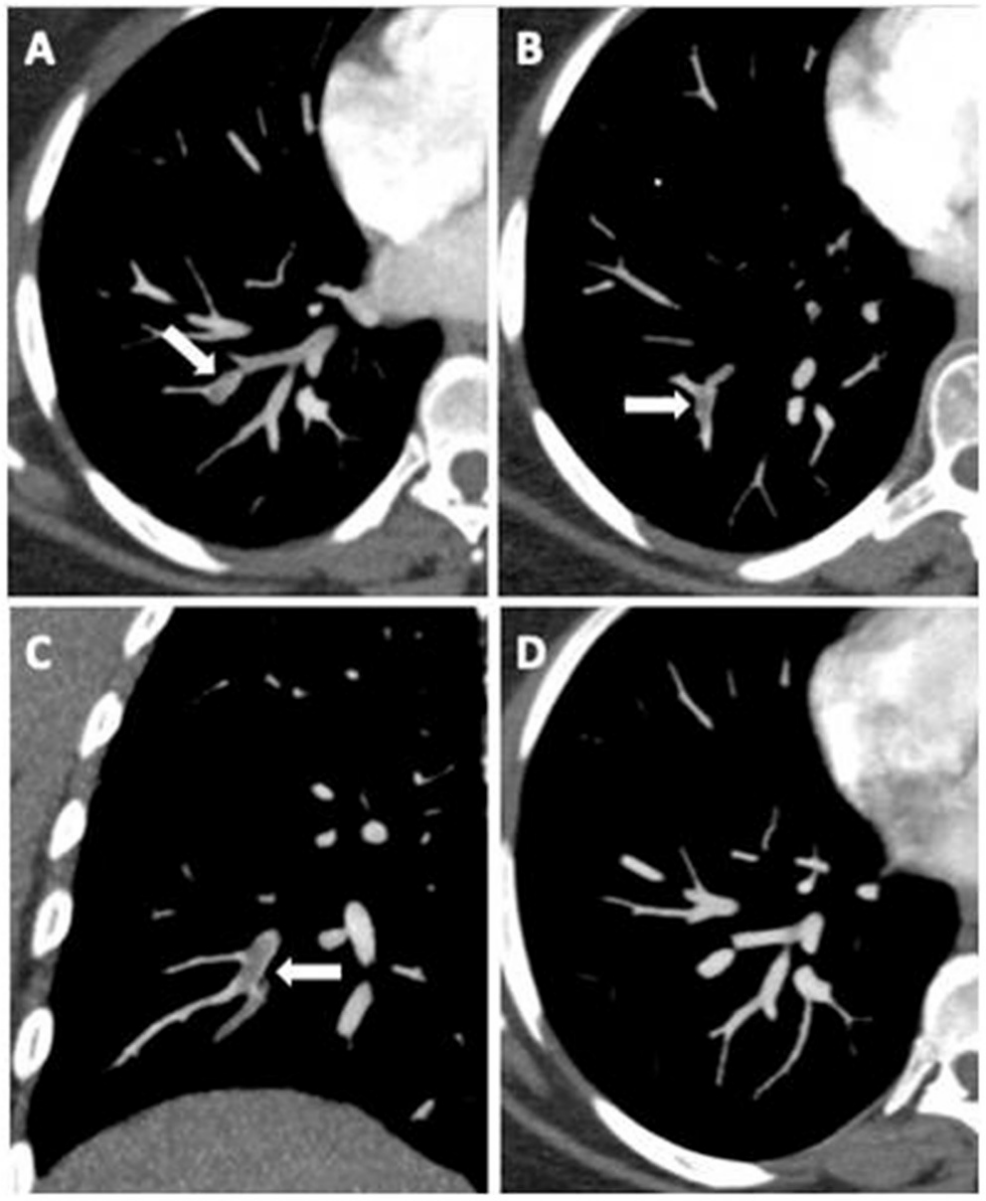
Axial CT images **(A,B)** and coronal multiplanar reconstruction **(C)** document partial centric and eccentric opacification defects in the right lower lobe basal segmental arteries (white arrows), suggestive of acute pulmonary embolism. Complete regression after anticoagulant therapy **(D)**.

## Discussion

In the present study we observed, for the first time, a significant higher prevalence of D/D ACE polymorphism in severe COVID-19 patients who developed pulmonary embolism as compared to those without thromboembolic complications. As compared with the general population, indeed, we observed a statistically significant difference as compared to COVID-19 PE+ patients, whilst no difference emerged as for comparison with COVID-19 PE− subgroup.

A recent study reported a higher prevalence of ACE D/D genotype in severe COVID-19 patients as compared to those with mild-disease, even though this association was dependent on the presence of hypertension comorbidity (16). ACE D/D polymorphism has been associated not only with hypertension, though also with obesity and diabetes, chronic conditions highly suggestive of high risk for COVID-19 infection, as well as for poor outcomes of the disease (17).

In addition, demographic studies have found in the racial variance of ACE I/D genotype a potential explanation of the different prevalence and outcomes due to COVID-19. In fact, the higher frequency of the D allele seems to perfectly match with the higher mortality rates observed in the African American population, as compared to Indians and White people, and in the European populations (particularly Italian, Spanish, and French) as compared to the Asian ethnic group (13, 14).

Likewise, a recent study reported an inverse correlation between ACE I/I genotype and both the prevalence and the mortality due to SARS-CoV-2 infection (18). Moreover, a meta-analysis demonstrated an association between the I/D allele frequency ratio and the recovery rate, though not for mortality rates (19). On the contrary, a study on people from Europe, North Africa and the Middle East demonstrated an inverse correlation between prevalence and mortality due to COVID-19 and the ACE D allele frequency (20).

In patients affected by acute respiratory distress syndrome (ARDS), previous studies have demonstrated an association between the D/D genotype and incidence, morbidity and mortality risk (21–24). In addition, SARS patients with an ACE D/D genotype disclosed a more severe degree of the disease, with the frequency of D allele higher in the hypoxemic group (25).

ACE D/D genotype has been suggested as a susceptibility marker of thrombosis. In fact, ACE D/D homozygosis has been associated to thromboembolism occurrence in subjects actually without predisposing factors and traditional thrombophilic alterations in other disease (26).

In the present study we did not find any significant difference of serum D-Dimer levels between COVID 19 patients either with or without pulmonary embolism. This finding could be related to the small number of patients recruited in the study. In contrast, the increase in C-reactive protein observed in patients undergoing to pulmonary embolism suggests the crucial role of inflammation in the pathogenesis of thrombotic complications in patients affected by SARS-CoV 2 infection.

In conclusion, the results of the present study let us hypothesize a genetic susceptibility to thromboembolism occurring in COVID 19 disease. ACE D/D polymorphism linked to higher levels of both ACE and angiotensin II could represent a genetic risk factor, although studies recruiting larger cohorts of patients are needed.

## Data Availability Statement

The data analyzed in this study is subject to the following licenses/restrictions: available after evaluation of the specific request and need. Requests to access these datasets should be directed to cecilia.calabrese@unicampania.it.

## Ethics Statement

The studies involving human participants were reviewed and approved by Ethic Committee of AORN Ospedali dei Colli and University of Campania Luigi Vanvitelli. The patients/participants provided their written informed consent to participate in this study.

## Author Contributions

The study design and conception was made by CC and AA. CC and GF were responsible for the whole content of the study and contributed to the draft of the manuscript. AC and VD managed data collection. PP was responsible for the data handling, statistical analysis, and data interpretation. SG performed and analyzed tomographic pulmonary angiography. All authors have read, written and approved the final version of the manuscript.

## Conflict of Interest

The authors declare that the research was conducted in the absence of any commercial or financial relationships that could be construed as a potential conflict of interest.
