# Supplementary figures and images for: ACE Gene I/D Polymorphism and Acute Pulmonary Embolism in COVID19 Pneumonia: A Potential Predisposing Role
Source: Front Med (Lausanne). 2021 Jan 21;7:631148. doi: 10.3389/fmed.2020.631148 (PMC7874110; doi:10.3389/fmed.2020.631148)

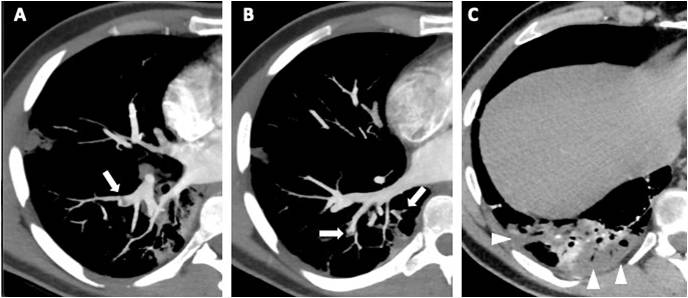

Supplement: Supplementary file 1 [file Image_1.JPEG]
