# Supplementary material for: ACE Gene I/D Polymorphism and Acute Pulmonary Embolism in COVID19 Pneumonia: A Potential Predisposing Role
Source: Front Med (Lausanne). 2021 Jan 21;7:631148. doi: 10.3389/fmed.2020.631148 (PMC7874110; doi:10.3389/fmed.2020.631148)
Supplement: Supplementary file 2 [file Table_1.DOCX]

Supplementary Material

## Supplementary Figures

**Supplementary Figure 1.** Axial CT images show partial centric and eccentric opacification defects, attributable to acute pulmonary thromboembolism, in the lumen of several right basal segmental arteries (white arrows in A and B) and infarct areas without enhancement in the corresponding lower lung lobe parenchyma ( white arrowheads in C).
